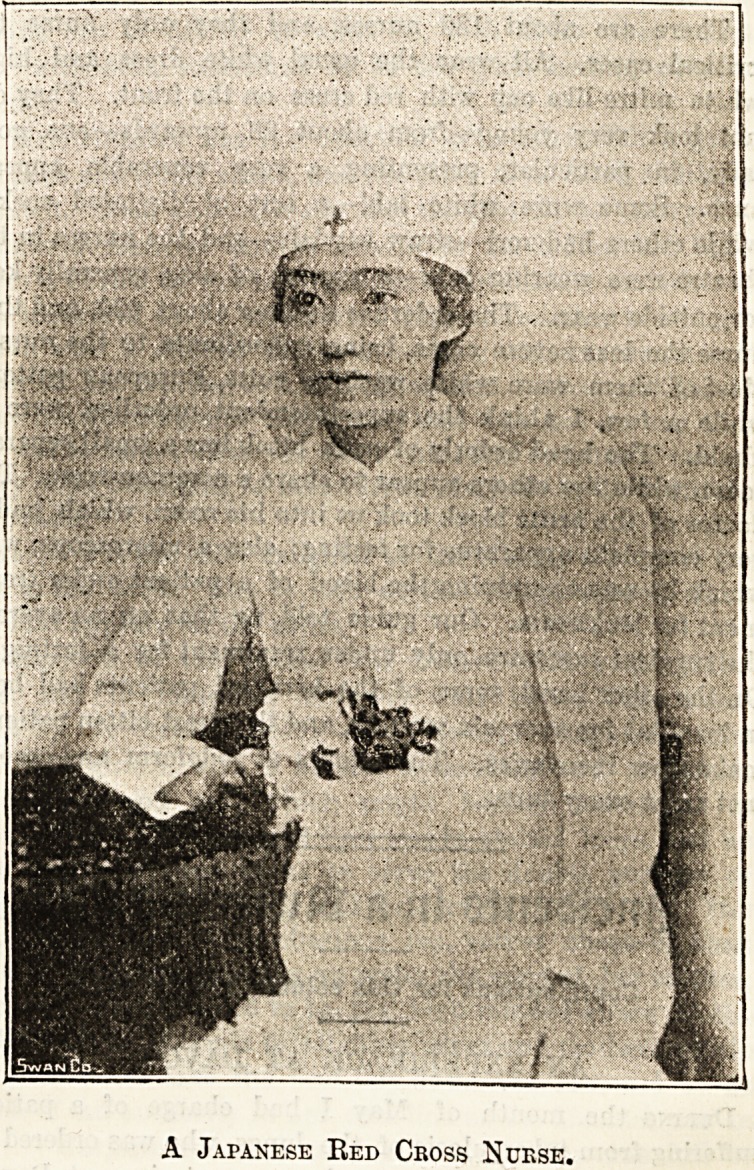# The Hospital. Nursing Section

**Published:** 1905-08-12

**Authors:** 


### The Hospital.

**Nursing Section.**

Contributions for this Section of "The Hospital" should be addressed to the Editor, "The Hospital" Nursing Section, 28 & 29 Southampton Street, Strand, London, W.C.

No. 985.—Vol. XXXVIII. SATURDAY, AUGUST 12, 1905.

### Notes on News from the Nursing World.

### JAPANESE NURSES AND ORDERLIES.

The English nurse who sends us an account of her visit to a Japanese Red Cross hospital in Tokyo, mentions that in this vast building, which accommodates between four and five thousand patients, there are about a hundred and thirty female nurses. Apparently there is no age limit in the Japanese Army Service for our contributor states that few of the nurses looked very young, while one presented a distinctly venerable appearance. They nurse only the critical cases, the others being under the care of six hundred orderlies, who are, of course, the subordinates of the nurses. That this system works well in Japan is beyond doubt, but English orderlies have not yet had the opportunity of showing themselves so capable as the Japanese appear to be.

### AN INQUIRY INTO POOR-LAW NURSING.

The determination of the Government to advise the King to issue a Royal Commission to inquire into the subject of the administration of the Poor-law, may possibly involve the non-appearance of the order which Mr. Gerald Balfour stated the other day was in preparation. It is true that the Commissioners may occupy two or three years in pursuing their investigations, and that even after the report is published there may be a considerable interval before legislation on the lines recommended is attempted. But the Commissioners will probably include the treatment and nursing of the sick in the scope of their inquiry, and, with all respect for the conclusions of the Departmental Committee, we think that a representative Commission, including, at any rate, one of the principal hospital matrons, will be better able to look at the nursing question from every point of view, and to make recommendations which would be for the advantage of the patients in the workhouse infirmaries and for that of the nurses employed. Although the system of nursing at the larger workhouse infirmaries has enormously improved in recent years, there is still great need in many of the smaller institutions for reforms which cannot altogether be effected by any order of the Department. In the interests of nursing, therefore, we shall warmly welcome the appointment of the Commission if, as we hope and anticipate, it includes persons who have a wide knowledge of all branches of work under the Poor-law.

### THE AGE LIMIT IN PRACTICE.

The Driffield Guardians have just been informed by the Local Government Board that in making future appointments it would be well not to select any person as nurse or assistant nurse who is not 21 years of age. The age for starting Poor-law nursing might, with advantage, be raised to 23. But while we think it most undesirable that very young women should be allowed to commence nursing in either hospitals or workhouse infirmaries, we are of opinion that the time fixed for retirement is often unnecessarily early. For example, Miss Sidney Browne is shortly relinquishing her post as matron-in-chief of Queen Alexandra's Imperial Military Nursing Service, primarily because there is an absurd age limit of 55. The vigour and ability with which Miss Browne discharges her duties supply a sufficient argument why the limit should either be altered or exceptions made. There is no reason whatever so far as physical capacity is concerned why Miss Browne should not be matron for another ten years.

### THE UNTRAINED NURSE AS MIDWIFERY PUPIL.

A fortnight ago we published a letter from a medical man who has been in the habit of taking trained nurses as pupils and preparing them for the examination of the London Obstetrical Society in return for their services. His experience up to a recent date had been that the arrangement was an advantage to him and a great help to those nurses who could not afford to pay a large fee for instruction. But, in order to oblige a former pupil, he consented, in an evil moment, to receive as pupil a young woman who was not a trained nurse. Our readers will remember what followed;—how, after sending her up for two examinations at which she failed, and helping her with money as well as advice, she at length passed her examination, and, having passed it, turned round on her instructor, gave herself airs, became impertinent, quarrelled with her teacher's qualified assistant, and set up in opposition to her teacher. We cannot imagine that she will obtain many pupils, because, apart from the fact that, as one of her instructor's pupils wrote last week, he is recognised as a most excellent coach, such unprofessional conduct generally brings its own punishment. But, as "M.D." points out in our issue to-day, the incident reveals a state of affairs that proves the difficulty which professional men of the present day have to encounter in dealing with women whose education and abilities are unsuited to the work they have associated themselves with. We agree that a principle of the Midwives Act is to allow the benefits of skilled nursing, under medical supervision, to the poor lying-in woman; and we believe that the purpose of the Act will be thwarted to a considerable extent if untrained nurses of the type described by "L.R.C.P." are certificated to any large degree under its provisions.

### FOLKESTONE CORPORATION AND THE MATRON OF THE SANATORIUM.

Following the complaint that, in the supposed interests of economy, the food of the nurses at Folkestone Sanatorium has been reduced so that the cost of living for each is now only 11¾d. per day, there was an extraordinary discussion last week at the monthly meeting of the Town Council. The new matron having resigned, it was recommended at the meeting that another applicant for the post should be interviewed, whereupon several members of the Corporation seriously urged that the new matron must either be a married woman or be prepared to marry the gardener. One of the opponents of this grotesque proposal, covered it with ridicule by suggesting that it might be added to the resolution that the medical officer be discharged and a new one appointed who should be compelled to marry the kitchenmaid; and in the end it was rejected. But so long as there are members of the Folkestone Corporation who think that the matron of the isolation hospital should be a person willing to marry the gardener, we do not wonder that there is friction in respect to the nursing arrangements.

### NURSING ASSOCIATIONS IN NATAL.

The formation of a Nurses' Association in Natal, which was announced in our columns some weeks ago, does not appear to be widely known beyond Maritzburg, where the movement originated. But a Durban correspondent, who is a fully-trained nurse, shows in a letter which we publish to-day that there is an opening for such an organisation, if only it be properly managed. Her experience of existing organisations in South Africa is not, however, calculated to inspire confidence in new schemes. As qualified nurses are advertising for engagements in the Natal papers, it is evident that newcomers are not wanted. We note that our correspondent regards five shillings per day as a reasonable payment on the part of poor people who require the services of a district nurse. If so, poverty in Natal must be of an entirely relative character.

### ROTHERHAM INFIRMARY NURSES AND RATIONS.

At the usual fortnightly meeting of the Rotherham Guardians a long discussion took place in consequence of a lady member of the Board being accused of advising the nurses at the infirmary who expressed dissatisfactian with the food to state their case to the male members of the Board. The lady in question in giving her version of the matter said that when she saw the nurses at dinner they complained of the tea, butter, and milk. She admitted that she recommended them to repeat the complaint to "the gentlemen Guardians," and she added that one of the nurses observed, "We never see them," whereupon she rejoined, "You go to confession," and the nurse said that she did. We do not understand why the question of confession was dragged into the controversy. But as to the point at issue, if in the present condition of affairs, nurses of a Poor-law Infirmary have reason to be discontented with their food, the proper course is to tell the superintendent nurse, who in her turn should report the matter to the master. In the event of his failure to take notice, an appeal to the Guardians in their collective capacity should be made, but not to individual members either male or female.

### LACK OF NURSES IN SOUTH DAKOTA.

With reference to our intimation on July 29th that there is a lack of nurses in some of the smaller towns in the north-west of America, we have received a number of letters from English nurses anxious to supply the demand. It is highly probable that before now the medical men in Idaho Falls have obtained the help they require, and that at Wessington Springs the nurse who was so badly wanted has been secured. We certainly do not advise anyone to go out to South Dakota on speculation, but a letter addressed to the principal medical man at either of the towns in question will doubtless elicit the information desired.

### MIDWIVES IN SURREY.

An interesting first report upon the operation of the Midwives Act in Surrey has just been published by Dr. Seaton, the medical officer of health for the county. The medical officers of health for the thirty-four urban and rural districts of the county have been appointed to perform the duties of midwifery inspection within their several districts, and they are called upon to report upon the midwife herself, upon her place of residence, and upon her mode of practice. The total number of midwives originally reported amounted to 334, but of these seven have died and ten have gone away, reducing the total to 317, of whom 102 have definitely announced their intention of giving up practice, presumably from inability to conform to the provisions of the Act. This will reduce the number to 215, in a county population estimated at 550,000, leaving one midwife to about 2,560 of population. In one urban district of the county, that of Ham, no midwife is to be found; and Dr. Seaton mentions that, in Midland and Northern counties, the proportion is said to be about 1 to 1,500 of population. In the present imperfect state of information, Dr. Seaton is of opinion that about 245 of the reported midwives come under the 1905 class, to which the provisions of the Act at present apply, and only 72 to the 1910 class, but this estimate may hereafter be found to require revision. Of the 245 not more than 104 can be classed as quite satisfactory, while there are 39 who might be considered as fairly satisfactory, and 102 as unsatisfactory; the last forming a class which it will be the aim of the medical officers gradually to abolish. Besides the 102 who have declared their intention of giving up practice, 50 others have expressed themselves as being very doubtful. Regarded as an account of the beginning of an organisation the report seems to be fairly satisfactory, but it may be doubted whether the number of well-qualified midwives likely to remain will at first be sufficient for the legitimate wants of the population, and whether general practitioners will not be inconvenienced by demands for their assistance which it will be difficult to refuse, and for which it will be impossible to obtain adequate remuneration.

### MISS THOROLD'S SUCCESSOR.

The Governors of Middlesex Hospital have lost no time in choosing a successor to Miss Thorold. The new lady superintendent is Miss Eleanor Caroline Vernet, who since June 1899 has been lady superintendent of the National Hospital for the Paralysed and Epileptic in Queen Square, Bloomsbury. Miss Vernet was herself a paying probationer at the Middlesex Hospital for a year, and in 1893 she entered the National Hospital as staff nurse. In 1895 she was promoted to be night sister, but relinquished that appointment in 1896 in order to take up the duties of sister at Middlesex Hospital, a post which she held until she was selected as head of the training school at the National. In an interview with Miss Vernet, which was reported in our issue of May 30, 1903, the new matron of the Middlesex Hospital expressed herself strongly in favour of giving late leave rather freely, because she holds the opinion that nurses ought to have plenty of amusement. She attaches great importance to the possession of tact, which, in fact, she considers the most indispensable quality of a nurse.

### THE RETIRING MATRON OF THE SAMARITAN FREE HOSPITAL.

There were two interesting ceremonies last week in connection with the resignation of the popular matron of the Samaritan Free Hospital. On July 26th Miss Butler received a handsome present from the lady visitors of the hospital in the shape of a dainty white purse filled with gold. The Hon. Lady Leigh made the presentation, and said that the lady visitors to the hospital had deputed her to present it to Miss Butler as a small token of the great esteem in which they hold her for faithful and devoted service for the past twenty years. Understanding that Miss Butler is thinking of taking a trip to South Africa the ladies who made the presentation begged her to use the money for her passage. On Monday last the sisters and nurses, together with Miss Royde Smith, the lady secretary, invited Miss Butler to a farewell tea, after which they asked her to accept from themselves and many of the past sisters and nurses as a token of their affection and esteem, a gold signet ring, with her monogram, bearing the inscription inside " S. F. H., August 1905." At the same time they gave her a large leather purse with silver mounts and clasp. Later on in the day the servants of the hospital presented Miss Butler with an umbrella mounted in silver.

### GENERAL BULLER ON THE WORK OF NURSES.

Speaking at the opening of a bazaar in aid of the local district nurses' fund, which was held in the grounds of Miskin Manor, Glamorganshire, Sir Redvers Buller expressed the pleasure which it afforded him to do a little to aid the excellent work which the function was intended to assist. Amid much applause General Buller asked, whether "there could be anything which appealed more to anyone either living on hill or in beautiful valley, in humble home or great, than the work of women who devote their service to the soothing of the bed of suffering or of death ? " There was a large attendance, and a handsome sum was realised for the benefit of the district nurses' fund.

### MISS NIGHTINGALE AND VACATION SCHOOLS.

At the opening last week, for the fourth successive year, of the Vacation School in connection with the Passmore Edwards Settlement in Tavistock Place, Mrs. Humphry Ward, to whose initiative the school is due, stated in her opening speech that among others who had given the movement special encouragement was Miss Florence Nightingale, who had promised a contribution. Miss Nightingale, with her knowledge of the needs of thousands of little people when they are supposed to be enjoying their holiday, recognises, we have no doubt, not only the absolute merits of this particular vacation school, but also the possibilities of development which nothing but a steady flow of subscriptions can, under present circumstances, render practicable.

### A DEMONSTRATION ROOM FOR A NURSES' HOME.

In memory of his first wife and only daughter, Mr. John Rees Robertson, of Toronto, has undertaken to build a new Home for the nurses of the Hospital for Sick Children in that city. The cost is to be 75,000 dollars; the plans have been adopted after a thorough inspection of most of the homes in the United States and the advice of several experienced superintendents, including Miss Nutting, of Johns Hopkins Hospital, Baltimore, Miss Brown, of Boston City Hospital, and Miss Brent, lady superintendent of the hospital to which the Home is to be attached. The building is to contain on the ground floor a large lecture hall, a reception-room, a library, and writing-rooms. It will accommodate 60 nurses, and in addition to a general kitchen, there will be a diet kitchen for the instruction of the nurses, under the supervision of an expert in domestic science. Moreover, there will be a large demonstration-room in the Home, so that the inmates may receive the necessary instruction before entering the wards. Special study rooms will be provided on each floor, and in the centre of the building there will be a gymnasium and a roof garden. It is hoped that the Home will be ready for occupation by October 1st next year.

### A BRIEF HOSPITAL EXPERIENCE.

A society paper announces that the daughter of a peer, who recently entered a children's hospital in London, has given up all idea of training, though it does not know whether she " decided that the nurses' uniform did not suit her complexion, or whether the glamour of hospital nursing evaporated on closer inspection." " Society," it adds, " is the better for the failure, since she is really one of those charming young people it cannot spare."

### OVERTRAINED AMERICAN NURSES.

The tendency in America towards the overtraining of nurses is exciting protests in influential quarters. The *St. Paul Medical Journal*, while freely conceding that the nurse should attain all possible skill in the performance of those duties which fall within her province, objects to her education along the line of diagnosis, pathology and treatment. Our contemporary maintains that knowledge of these subjects does not enhance her value as a nurse, and is likely to do her nothing but harm.

### The Nursing Outlook.

"From magnanimity, all fear above;
From nobler recompense, above applause,
Which owes to man's short outlook all its charm."

### VILLAGE AND MENTAL NURSES; LICENSED HOMES AND INSTITUTIONS.

Two points appear to have specially exercised the minds of the Members of the House of Commons Committee on the Registration of Nurses—*i.e.* Village Nursing and the Control of the Supply agencies.

In order to understand the first it is necessary to remember that a member who represents a county naturally hears most about nursing from the villages and smaller communities. For this reason, possibly, the position of the village nurse, if we may judge by the number of questions asked on this point by members of the Committee during its sittings, was regarded by them as specially important from the public point of view. In their Report they give expression to this feeling by declaring that it should be the duty of the Nursing Council within four years of the passing of any Registration Act to submit a Report to the Privy Council as to a separate register of nurses of a lower standard than the registered nurse. We entirely favour the view expressed by the Committee in this recommendation because any improvement in nursing matters in this country must be gradual and slow if it is to prove advantageous to all the interests involved. Village nursing is practically in its infancy and it will be time enough to consider what steps, if any, should be taken in reference to this matter when the larger questions involved have been thrashed out and settled.

Meanwhile the want of some representative and voluntary organisation which includes all the interests concerned is generally recognised, and an incorporated society with the co-operation of the nurse-training schools, or at least of the Managers of the more enterprising and intelligent of such schools, might usefully be established without delay. Such a step would facilitate the affiliation of the nurse-training schools and the co-ordination of all the elements which must be brought together in order to secure the maximum result for good from the changes in nursing which are impending.

Mental nursing has long occupied an unsatisfactory position in England especially. The Medico-Psychological Association has laboured assiduously to improve the standard of mental nursing and its efforts have led to important results, especially in Scotland, and latterly in England. The House of Commons Committee have recognised the claims to registration of mental or asylum nurses. They are of opinion that a separate register of "Registered Asylum Nurses" should be kept by the Nursing Council, to which should be admitted the names of nurses who have served for not less than three years, in not more than two asylums, have received a certificate of the Medico-Psychological Association, and can produce satisfactory certificates of good character. No doubt such a register would prove an assistance to those who have to find and employ mental nurses. The difficulty at the present time is largely due to the circumstances, that many certificated hospital-trained nurses have a dread of mental cases, because they have had little or no experience in handling them. An adequate supply of efficient mental nurses would therefore be a great boon to the medical profession and to the public. But if the present want is to be supplied the mental nurse must have an all-round training. Experience shows, that mere residence in an asylum, as an attendant upon the insane, need not, and often does not constitute, an efficient mental nurse.

The last four paragraphs of the Committee's Report deal with the important subject of the treatment of nursing homes and institutions. Nursing Homes are defined as all homes and places conducted for profit wherein patients are taken for treatment. Nursing Institutions are described as societies or bodies which supply nurses to the public. The committee consider the licensing of such homes and institutions, by the county or county borough authority in whose area the home or institution is situated, to be highly desirable. The issue of a license to any such establishment is to be conditional upon a guarantee on the part of the proprietors that when any nurse employed or sent out by any such establishment is not a registered nurse this fact shall be definitely stated to the employer and presumably to the medical attendant in charge of the case. These recommendations accord with the plan we have continued to advocate in regard to these establishments. It is an undoubted fact that directly the local authorities control the sources of supply of nurses to the public, from that moment the death-knell of the pseudo-nurse will be sounded. It has no doubt been a great temptation in the past to the least reputable owners of private adventure homes at any rate, whenever the demand for nurses has become great, to send out women, who they must have known, or ought to have known, could not be efficient nurses, for the reason, that they have never had any real opportunity of being practically trained for the work.

It is too early to determine exactly what will be the effect of the Committee's Report on Nurse Registration, but one result of it should be to secure the licensing and registration of Nursing Homes and Institutions which supply nurses to the public without any further delay.

### Medical Electricity and Light Treatment.

By Kate Neale, Sister-in-Charge of the Actino-Therapeutic Department, Guy's Hospital.

### IV.—RADIUM. VIBRATORY MASSAGE. ELECTRIC CAUTERY.

### Radium.

Radium has excited so much popular interest in the past few years that you are probably already familiar with many of its properties. It is a chemical body obtained in small quantity from certain Bohemian mines, where it occurs in the ore known as pitch-blende. The point of chief interest to us and one that confers on the substance medicinal value, is that it gives off constantly and spontaneously certain rays which have been proved to exercise a curative effect on some diseases. A further peculiarity is that the radium maintains itself at a temperature a few degrees higher than the surrounding air. It is employed in medicine in three or four different forms. The variety most frequently used is radium bromide, a purple-coloured powder, while another compound (with barium) is know as radium barium. This, too, is a powder, but of a yellowish-white tinge. Other forms are French radium and radium nitrate.

The activity of radium is greatly impaired by moisture, and it is therefore often kept in small sealed glass tubes. These are two to three inches in length and of the diameter of a goose-quill. You must use the greatest care to avoid any breakage, for the amount of material inside, small though it is, costs many pounds.

*How to Treat.*

The application of radium to a diseased surface is a very simple procedure, so simple that it can be left quite safely to the patient, and in some cases, as we shall see, this arrangement is to be preferred. The radium is shaken down to one extremity of the tube and that end pressed lightly against the spot to be treated. You need make no preliminary preparation, unless it be to cleanse the part from dried scabs, etc., and no precautions have to be taken to render the part bloodless, as in the case of Finsen light. After the tube has remained in position for half an hour (the usual duration of a single treatment) a dressing, such as spread boracic ointment, may be applied, and the patient is finished with. The application should be repeated every one, two, or three days, according to the doctor's instructions, until cure is completed, or, at any rate, no further improvement obtained.

At the end of a treatment, and before putting the tube away, remember always to carbolise it, for fear of carrying infection from one patient to another. Some makers send out their radium in vulcanite cases, with a talc window at one end through which the rays pass. If such be the form you use, be careful in carbolising to prevent any liquid running under the edge of the talc and wetting the radium.

A frequent site for treatment is the interior of the nose or mouth, and in these cases you will be wise to leave the application in the patient's hand entirely. It is difficult for you to know exactly when the tube is on the affected area, whereas he will recognise the fact at once by sense of touch. Impress on him, however, the importance of applying the end of the tube where the radium is collected. I remember a patient who placed the upper end of the tube inside the nostril, while the powder, of course, fell to the lower end, which she was holding in her hands.

*Diseases Treated.*

The number of conditions at present known to be influenced by radium is small. Rodent ulcers are perhaps more often benefited than any other disease, but good results have also followed applications to lupus patches. The treatment is especially useful when the mucous cavities (nose or mouth) are affected, and the ulcerated spot is difficult to get at by any other means. Advanced cases of malignant disease which are already beyond the surgeon's aid are sometimes put, as a last hope, under a course of radium treatment.

### Vibratory Massage.

One of the more recent developments of massage takes the form of an electrical apparatus for applying a rapidly vibrating electrode to the patient's muscles. Although no actual current is given, the use of the electrode is accompanied by a peculiarly bracing sensation. This treatment is know as *vibratory massage*, and enjoys considerable popularity, especially in those cases where ordinary massage would be serviceable. It cannot be regarded as a serious rival to its elder sister, but is useful rather as an adjunct to this latter than as a substitute, and accordingly you will often find the two prescribed together.

*Apparatus.*

The apparatus is depicted in fig. 5. It consists of an electric motor A, worked by means of the current

Fig. 5.—Apparatus for Vibratory Massage.

from the main, and ending on the left in a flexible tube about three feet in length. The motor is started by turning the switch B, and its speed regulated by sliding the button C along the slot DD. The free end of the flexible tube is attached to the ball vibrator B V, which consists of a handle terminating in a metal globe. In fig. 6 the vibrator is seen on a larger scale. The globe, some $3\frac{1}{2}$ inches in diameter, rests on a collar on the surface of which a single line is engraved, and below this comes a short metal neck carrying the numbers 0, 1, 2, 3, 4, 5, 6, 7, 8 at its upper end, and a milled screw-head at its lower. This neck can be rotated by means of the milled head, and each of the numbers 0 . . . 8

FIG. 6.—VIBRATOR.

brought in turn beneath the engraved line. This arrangement is to intensify or diminish the vibrations in the ball in such a way that when the number 0 is opposite the engraved line the vibrations are very feeble, but as the higher numbers are successively screwed round to coincide with the line, the vibrations grow stronger until at 8 they are very forcible indeed. Below the milled head is the handle of the vibrator; the globe, which is the part applied to the patient, is perforated by two or three holes, into which special terminals can be screwed when it is desired to localise the application to an area of skin smaller than the globe itself would cover.

*How to Treat.*

Bare the part of the body that is to be treated, whether it be a limb or the abdomen. Switch on the current from the main and turn the handle, B. This will start the motor and its rate you regulate by the button and slot C, DD. Then, having adjusted the force of vibration by means of the milled head in the manner described in the preceding paragraph, grasp the vibrator (ball downwards) in your right hand, holding it as you would a dagger, *i.e.* your thumb uppermost, and not as you would a tennis raquet or a golf club. Pressing the ball firmly against the skin, move it up and down in much the same fashion as in labile applications. It is always better to treat off the end of the ball, rather than from the side, as in the latter case the full force of the vibrations is not felt. When the weather is cold, warm the vibrator in front of the fire before use; for this purpose the flexible tube can be readily unscrewed from the motor. A single treatment lasts from 10 to 20 minutes.

*Diseases Treated.*

The conditions that are usually treated with vibratory massage are much the same as those for which ordinary massage is ordered, *e.g.* paralysis of muscles, injuries to nerves, etc. The pain arising from chronic peritoneal adhesions may be relieved by the vibrator, while in chronic constipation its effect has been tried by local applications to the abdomen.

ELECTRIC CAUTERY.

Though the electric cautery is an instrument you must never use yourself—the surgeon alone must apply it—you should be able to fit one up in readiness for an operation. This is a very simple matter, but you will learn how it is done better with an instrument before you than from any written instructions. It will, therefore, suffice to say that a cautery consists of a battery of cells, or of a so-called "accumulator" in which electricity has been previously stored, the current being led by two wires to a cautery-needle provided with a platinum point. On making the circuit through the needle, the latter becomes heated to a red or white heat according to the strength of current used, in exactly the same way as the carbon filament of an electric light glows as soon as the current is switched on. The current is regulated by a "resistance," consisting of a coil of wire so arranged that a greater or less number of turns can be put into the circuit by means of a movable handle, or sliding terminal. We saw in Chapter I. how the greater the resistance the weaker was the current, and it follows that the more resistance you put into the cautery the less will be the heat generated in the needle. As a rule a dull red glow is enough for surgical work.

When the apparatus is finished with, care must be taken to prevent the terminals lying in contact, as this would rapidly exhaust the battery or accumulator. You should therefore unscrew the wires and coil them up for further use.

### The Nurses' Clinic.

### THE DISPENSARY. BY A CERTIFICATED DISPENSER.

### EMULSIONS.

THERE is a certain class of mixtures which require more skill and care in compounding than most others: these are called emulsions. An emulsion is a thick, milky-looking mixture containing an oil or resin in suspension, or a heavy powder such as bismuth. The usual agents used are gum acacia, tragacanth, yolk of egg, or an alkali. Mucilage can be kept either ready mixed, as the mucilages of tragacanth or acacia, which are official in the British Pharmacopœia, or the equivalent of dry powder may be used. Some substances when rubbed up with water form perfect emulsions, as the gum-resins ammoniacum, asafœtida, and myrrh; these are called natural emulsions, and the explanation is that each contains as much gum as will suspend it on the addition of water. If ready-made mucilage is used it must always be fresh; mucilage of acacia does not keep well after the bottle containing it has been uncorked. If powdered acacia be used, about half the amount of mucilage ordered will be required, as 60 grains are contained in about 144 minims of the mucilage. Pulv. tragacanth does not, as a rule, form a very good emulsion; it is better to use the pulv. trag. co. Mucilage and an alkali should not be used together, as an emulsion formed by one will often be separated on the addition of the other; if it is unavoidable the alkali should be added last and should be very dilute. Syrup and preparations containing spirit should be added last. Spirits of wine, concentrated acids, and tannin should all be added in combination with as much of the vehicle as possible. All solids to be dissolved in emulsions should be separately dissolved in the water and then mixed in; if mixed with the emulsifier they may cause separation. Glycerine, like

### THE NURSES' CLINIC—*Continued.*

an alkaline salt, is a disturbing agent when another emulsifier is present. Borax is a good emulsifier, but when mixed with acacia it forms a jelly after a few hours. Mucilage forms a better emulsion with castor oil and copaiba, but an alkali is best for oil of almonds. Copaiba is best done with equal quantities of liq. potassæ and water. Lime water and linseed oil or olive oil in equal quantities form an excellent emulsion known as Carron oil, which is much used for application for burns or scalds.

There are two different ways of making emulsions; one is by rubbing the substance in the mortar with the emulsifier, the powders being first rubbed up dry together and the water added slowly until a perfectly smooth creamy mixture is formed. The second way is what is known as "Forbes's Method" for emulsifying essential oils, which consists of putting about 20 grains of acacia to each ounce of oil into the bottle, adding the oil little by little and shaking well, then adding the water also by degrees; the object being to split up each particle of oil and to get it covered with the mucilage to such an extent as to prevent its again uniting with the neighbouring globules.

An essential oil is a thin volatile oil produced by distillation, as turpentine or eucalyptus, and which does not leave greasy marks; the fixed oils are those which are obtained by expression and are thick and sticky; they are sometimes called "expressed oils." Warm water must never be used in a mixture containing volatile oils. If a liniment containing turpentine and yolk of egg be ordered, the egg (which must be quite fresh) is cracked on the side of a glass and the yolk carefully separated from the white, a trace of which may spoil the emulsion; the yolk is then stirred in a mortar until quite smooth, and rather more than its own bulk of water is added, slowly stirring all the time, and when perfectly done it is strained through muslin into the bottle, and the turpentine added by degrees and well shaken, and when a perfect emulsion is formed the other ingredients may be added. It is important to strain the egg into the bottle to be quite sure that none of the white be present. This emulsion will naturally not keep long. Balsam of Peru also forms a good emulsion made in this manner with yolk of egg. Spermaceti is best made with egg after having been finely powdered, but it can be done with a little sugar and pulv. acaciæ, the water being gradually added with constant stirring. Ol. santali is usually made up with about twice its bulk of fresh mucilage.

According to the British Pharmacopœia, mist. ol. ricini should be prepared by mixing alternately and gradually castor oil and a mixture of undiluted orange flower water and cinnamon water with mucilage of gum acacia; but it may be found better, on the whole, to rub up the oil and mucilage together in a mortar and then gradually stir in the water; the method of doing it, however, is best left to the discretion of the dispenser; some find they prefer one way and some another. If a prescription occurs for an emulsion containing an oil and camphor, the latter must be dissolved in the oil, then rubbed up with the mucilage before adding the water. If there be no oil ordered the camphor should be finely powdered with the aid of spirit and proceeded with in the usual way. Tincture of senega is a very efficient emulsifier of fats and oils; five minims of the tincture will emulsify half an ounce of a fixed oil. Tincture of quillaia possesses the same power; the active principle of both is saponin; tincture of quillaia (Panama) is prepared by percolation of one part of the inner bark, coarsely powdered, with proof spirit to produce five parts. In a case where two volatile oils occur they should be put together and emulsified by Forbes's method as if they were one; or if one be fixed and the other a volatile oil, they must be emulsified separately, the one in a mortar and the other in the bottle, and afterwards carefully mixed and well shaken together.

Mucilage should not as a rule be united with iron preparations; Syrup Ferri Phos. Co., however, is an exception. If iron is ordered in an emulsion of oil it must be added very dilute, but in the case of a scale preparation, which must always be put first into a mixture, the difficulty may be overcome by the dispenser (with the physician's sanction) emulsifying the oil with carbonate of potash, which is a very good agent when no other emulsifier is present. In mixing an emulsion in a mortar the emulsifying agent should generally be taken first and the substance added to it, not the agent to the substance.

Resins should be emulsified with gum acacia in the mortar, the water being gradually added. A good example is the guaiacum mixture of the Pharmacopœia. It is a good plan, as has been remarked in another paper, to keep this preparation ready mixed, as it takes a considerable time to do in large quantities, and when kept in bulk the required amount may be quickly weighed out and combined with the vehicle. Resinous tinctures, as asafœtida, benzoin, cannabis indica, and others are better made up in the bottle with mucilage of acacia, which should be quite fresh; the mucilage is shaken up in the bottle with about three times its own weight of water, and the tincture added and thoroughly agitated, and the rest of the water added by degrees. It may be of interest to the reader to know that the cannabis indica, or "Indian hemp," mentioned above is the celebrated intoxicating "bang" or "hashish" of the Eastern nations. It is also known as "gunjah," or "ganja."

The mist. amygdalæ of the Pharmacopœia is a white emulsion, used chiefly as a vehicle for other medicines and as a basis for lotions. It is composed of sweet almonds (with their coats removed), sugar, and gum acacia, which are well rubbed up with water and then strained through muslin.

Liquid extract of male fern is sometimes very difficult to dispense. It is best done with egg, but it can be done with milk, fresh mucilage, or pulv. tragacanth.

### Visit to a Japanese Red Cross Hospital.

BY AN ENGLISH NURSE IN TOKYO.

The hospital, which is built on the pavilion system, has accommodation for about 4,300 patients, but at the time of my visit with another nurse only about 1,500 were being nursed within its walls. It stands high up in one of the nicest districts of Tokyo, and is surrounded by pretty, well-wooded grounds. On arrival I presented our permit to a very solemn looking personage, clad in white European garb, who was busily writing at a table just inside the principal entrance, and after a little confabulation we were conducted through some long, long corridors to a very bare little waiting-room attached to the new blocks. Shortly after a boy arrived, bearing two minute cups of Japanese tea, which he presented to us with profound bows. Before we had finished this refreshment in came a very courteous little army surgeon, a major, who had quite a good knowledge of English. We explained that we were English nurses much interested in his hospital, and asked permission to see some of the wards. He first piloted us across to the club house—a solid, brick building, in which lectures are given, entertainments held, and where the convalescents can play billiards, etc. A bright, cheerful room, painted pale green, with a gallery running round, and a large raised platform at one side, from which the head orderly was giving a blackboard lecture to some 50 other orderlies, all of whom were squatting on the floor, armed with pencils and note-books. Just beyond the club house is an open-air theatre, so that the convalescents are well catered for in the way of amusements.

### The Wards.

The officers are nursed in the older building, and have small single rooms, containing a bed of the Lawson Tait pattern, a small table, and a few other necessaries. The ordinary Tommies are nursed in wards constructed since the outbreak of hostilities, raised wooden buildings with iron roofs, boarded floors, and bare wooden walls. The ventilation seemed good, plenty of sliding windows and ventilators worked by pulleys, all of ground glass. Many of the wards contain 38 beds, arranged in double rows, not much space between, and certainly not arranged for bulky people. The beds are all wooden, sort of oblong, narrow boxes, raised about eight inches from the floor, with bamboo mattresses, small hard bolsters (suggestive of knapsacks), and the usual Japanese pitons (padded quilts), covered with unbleached cotton material. A few of the beds had bright scarlet blankets, but our guide said that these were chiefly reserved for the cold season. A narrow board, fixed to the head of each bed, serves as a table for medicine bottles, teacups, pipes, tobacco, books, etc., and of course the inevitable fan. Some of the patients had small photographs and pictures fastened to the walls, while others had scraps of heather or a few wild flowers in small bottles, and quite a number sported small glass bowls of gold fish. Dusting must be a delightfully simple matter—no lockers, no polished tables, no chairs with dust-catching corners, no screens, absolutely nothing but beds.

### Ailments of the Patients.

The surgical patients, many of whom were hopping round on crutches, looked particularly bright and happy. Several of them were suffering from the effects of frost-bite, one having had all his toes amputated, and another a hand. They all wear white kimonos with a small red cross on the left sleeve, those well enough to go outside the wards being provided with round white caps, also adorned with a red cross. A large proportion of the medical patients were undergoing treatment for beri-beri, or kakke, as the Japanese call it. Massage is given extensively for these cases, also galvanism. There were a few cases of malaria, also a sprinkling of pneumonias and a very small proportion of enterics. Outside each ward is a large black slab, giving details of the patients' diseases, also stating whether they are able to walk or require to be carried. Small wooden tags with patient's name, regiment, etc., are hung outside the ward doors, a corresponding tag being fastened to each bed.

### Critical Cases.

The critical cases are nursed in small two-bedded wards, furnished as simply as the larger ones, only that the beds are provided with green mosquito nets. We saw several men with beri-beri of the severe type, but were told that these were improving under treatment. One man had been operated on for cancer of the stomach, another had traumatic pleurisy —the result of a bullet wound—another had partial paralysis, caused by a bullet wound in the spine. We saw a case of meningitis, also an advanced tuberculosis. Enterics were very much in the minority. Dressings were being done in a sort of surgery, those patients unable to walk being carried across on stretchers. There was a large centre table covered with brown American cloth, on which stood various instrument sterilisers all bubbling away vigorously, large glass jars of corrosive sublimate, lysol and alcohol, also jars of gauze, cases of wool, etc. The instruments in use were lying in antiseptic solution, in covered metal trays. The dressers wear white overalls and appear to work with bare arms. Dressing mackintoshes are conspicuous by their absence, sheets of oiled paper being used instead. One poor fellow, with six bullet-wounds in the upper arm, was sitting like a statue, while the dresser probed vigorously. It must have been excruciatingly painful, for the man looked ghastly, and the perspiration streamed off his face, but he made no sound—Japanese pluck. The bandaging looked very neat and up-to-date.

### Dispensaries and Kitchen.

There are three dispensaries, the one we were taken into looked very trim and well-fitted. The medicine bottles have

A Japanese Red Cross Nurse.

metal caps, fastened by clips. There was a large pigeon-hole affair at one side, where the bottles and prescriptions from the various wards are placed, each ward having a special compartment. Our guide seemed specially proud of this arrangement. Dinners were just being served from one of the largest kitchens as we passed through. Large stone stoves, with big cauldrons for rice, bean-soup, etc. Wood is used for fuel. Rice is, of course, the principal article of diet, and of this each patient gets six "gos" per day. It is carried to the wards in small wooden boxes. The rest of the midday meal seemed to consist of small portions of dried fish, surrounded by rather weird-looking vegetables; this was arranged on small enamel plates. Special cases had a diet of bread as well. There is quite a large milk kitchen with a sterilising apparatus, the milk being distributed in small bottles (about 8 oz. size) with patent corks. Tea and rice-water appear to be the principal beverages. The patients get three principal meals daily.

**JAPANESE RED CROSS HOSPITAL**—*Continued.*

The Operating Theatre.

We did not see the principal theatre as an operation was going on, but we went through a smaller one in the new block. This has a perforated zinc table, heated by hot water, a fair-sized wooden cupboard with glass doors for instruments, and small wooden tables for lotion-bowls, trays, etc. The walls are plain, unpainted wood, and the floor rough stone. A patient who had just had both feet amputated for frost-bite, and also had an extensive bullet wound of the arm, was just being carried back to the wards. Sterilisers of various kinds are very much in evidence, but the sinks and basins are all in an adjoining room—no water laid on in the theatre itself.

The Nurses and Orderlies.

There are about 130 nurses, and they only nurse the critical cases. All wear the usual white dress and high, white mitre-like cap with red cross on the front. They did not look very young—from about 28 upwards—one good lady, in particular, presenting a very venerable appearance. Some wore white tabi—a sort of digitated sock—while others had zori—straw sandals—and the nurses in the theatre were wearing geta—a species of clog, generally kept for outside wear. The orderlies number about 600, and they nurse the less severe cases, being subordinate to the nurses. Most of them wore white washing suits, European pattern, while a few, I think the superintendent orderlies, were in khaki. The head orderly of each block has a small separate room, while the others appear to share a common room. The doctor of the acute block took us into his room, which had a very complete apparatus for testing; also a microscope, with which he was examining the blood of a patient under treat ment for leukæmia. Our guide told us that on an average the surgical cases are only under treatment for a fortnight. On the other hand, some of the beri-beri patients had been in hospital for over six months, and the frost-bitten patients make slow recoveries. The odours of iodoform and creolin met us at every turn.

### Incidents in a Nurse's Life.

**Contributions for this column are invited.**

### AN EXPERIENCE AT DAVOS.

During the month of May I had charge of a patient suffering from tuberculosis of the lungs, who was ordered to undergo 12 months' treatment in a sanatorium at Davos-Platz. We travelled by Folkestone, Boulogne, Basle, Zurich, and Landquart, reaching Davos about 36 hours after leaving London. Our destination was Schatzalp—1,000 feet higher than the town of Davos—reached by a funicular railway, where we found the sanatorium finely situated above the pines on a snow-clad mountain overlooking the whole valley or plateau of Davos, completely surrounded by snowy peaks, which in the bright sunshine formed a scene of dazzling beauty.

The building accommodated 150 persons, but at this time of the year when many of the patients were allowed to leave the heights for change of air and scene, there were only about fifty undergoing treatment. Our first introduction was to the spacious *salle-à-manger*, which was arranged like an ordinary hotel, with numbers of small tables, at which were seated groups of tanned, healthy-looking individuals, men and women, who all appeared to do full justice to the tempting fare provided. On the conclusion of the meal I asked one of the doctors where the patients were, as I supposed these must be their friends or relatives, and, moreover, I had not heard the sound of a cough, which one would naturally expect in a such a place. I was informed that, with the exception of myself and the medical staff, all the people present were patients, and that coughing being a habit which could be controlled, it was not allowed in the dining-hall.

This I found to be a fact, and during the whole time of my stay in the sanatorium I do not think I ever saw any evidence of the nature of the disease from which the patients suffered, though there were occasional mysterious disappearances, and I was aware that every patient carried a pocket-flask for purposes of expectoration.

The treatment commenced at 7 A.M., every patient being either sponged all over or ordered a bath at a certain temperature which was prepared by attendants. From this hour until 9 P.M. the time was mapped out, the programme, consisting of meals, exercise, and "the cure" following in regular rotation. Doing the cure was interpreted by lying on a lounge in a balcony, and like the other rules was carried out with clock-like regularity, nothing being allowed to interfere with the purpose for which the patients entered the building. The meals were frequent, and exercise was measured by zig-zag paths still higher up the mountain, there being numbered seats each time the paths turned. It was impossible to feel dull or find the days long or wearisome, the difficulty being to get a few spare minutes or time off, in which to visit the little town below, or take part in the pleasures peculiar to the place. Occasionally there were musical evenings in the recreation-room of the sanatorium, but late hours were quite against the rules. Unlike the sanatoria which I had visited in England, the patients did not sleep in the open air, but all bedrooms were fitted with hot-air pipes which might be put on or off at convenience, and even open windows were not compulsory, the doctor in charge explaining that they did not believe in such severe measures. He said that their results were good, the majority recovering after prolonged residence in the cold dry air of the mountains, but I found from inquiries that he was certainly taking the most favourable view, as I heard of several cases amongst the few present having to return there winter after winter, and I travelled back with a lady of 28, who, after 20 consecutive months' treatment in the sanatorium, was returning home as incurable. No doubt results are similar in our open-air sanatoria, as we know that the disease is only curable in its earliest stage, when it is most difficult to be diagnosed and taken in hand.

The town of Davos is rather overcrowded; houses and hotels all being built at one end of the plateau in close proximity, though there is ample room further along the valley where the Queen's Sanatorium is in course of erection, while others are being planned higher up the mountains. I cannot speak enthusiastically enough of the beautiful crisp air and bright sunshine, nor of the Alpine flowers which spring up in patches wherever the snow has disappeared—anemones, blue gentian, and tiny white crocuses—while as one descends the mountains, the valleys are a blaze of colour with bluebells and forget-me-nots. I soon discovered that my ideas of the beauty of the landscape by no means corresponded with the general views, the patients becoming weary of the never-ending scene of pine and snow. The staff of the sanatorium consisted of two resident and one visiting physician; two German-Suisse nurses who looked after the patients took temperatures and carried out special instructions; while the sterilising of the day and night expectorators and collecting of various specimens was done by a sort of *homme de chambre*, and all arrangements of a sanitary nature were most perfectly and systematically attended to.

### A Book and its Story.

### IN THE DAYS OF DISRUPTION.*

Miss Sarah Tytler's latest story, like every other that she has written, is wholly harmless and at the same time full of human interest. The characters move in a restricted area and are remarkable by reason of their individuality and moral worth rather than for any picturesque grace, for the exercise of which there is little space in the hardy life of a Scottish country parish. The date of "A Daughter of the Manse" is the early days of Queen Victoria's reign, and the motive of the story is concerned with the split in the Church of Scotland which ended in the formation of the Free Church by the disaffected members. How strong was the conviction that animated the members of different classes gathered together in Edinburgh to detach themselves from the State Church the following passage will show:—"All Scotland, Edinburgh especially, the scene of the deed was, in its crowded condition, tense with expectation. St. Andrew's Church was packed with ministers and elders, and every one who could put in the slightest claim to be present. . . . From the wilds of Orkney and Skye, from the clay beds of Caithness, from the drifted sands of Nairn, from the Braes of Mar, and the Braes of Angus, from the lofty ridges of the Grampians, and the deep hollows of the Teviots, from the herring and "haddie" fleets of Loch Fyne and Findon, from ancient universities like St. Andrews and Aberdeen . . . . from the capital itself, guarded by its couchant lion, from the wan waters of the borders they had come." The following description will give to those readers unfamiliar with a Scottish manse and the position of a Scottish minister, some idea of the likeness between them and an English country cure of the same date:—

"The Manse of Rowanden was not so ancient as the church; it was comparatively modern, while it was quite as large. It had been built at a time when the successors of Knox and Melville were still for the most part lairds' sons who had private patrimonies to add to their stipends. . . . This accounts for the fact that these manses, especially if they were in the country, were substantial, spacious country houses, the glebe lands attached to the livings affording room and material for pleasant garden grounds, masking the farm offices in the background until there was little to distinguish the minister's house from that of his kinsman, the lord of the manor. Times were changing, the ministers were less and less frequently drawn from the country-gentry class, whose sons had lived to see that neither wealth nor rank was to be won in the sacred profession, that unless they went into it from the higher motives which set at naught worldly ambition, it was idle to think that they could secure the power dear to men's hearts, the social influence and political importance which had been possessed by the fathers of the Church. The manses, comfortable and agreeable residences as they presented themselves, began to be somewhat of a burden and a strain on the less refined habits and more straitened means of their occupants."

The particular parish in Scotland in which the manse was situated is described as being on the border-land, where the Highlands and Lowlands meet; in the foreground were green pastures and yellow cornfields, leafy plantings giving welcome relief to the eye, and the blue mountain ranges distance and dignity to the otherwise homely scene. No railway was near, and the local coach did not come within six miles of the remote village of Rowanden, presided over by the Rev. Archibald Menzies, D.D. A man of simple and unambitious nature, "He was content with Rowanden Kirk and Rowanden Manse and their minimum income, which any well-to-do tradesman might have despised, meted out to him according to the market price of so many chalders of grain, with his handful of more or less humble parishioners—who yet filled the tiny ancient kirk with its stone roof and its great square tower to overflowing—as his father had been before him. . . . The natives were like the country; their wholesome country breeding and lack of sophistication constituted their chief charm, while they had also their two sides—their honest country bumpkin side and their brutal Caliban side—as was known to his sorrow by Dr. Archibald Menzies, the learned Doctor of Divinity and meek and pious man of God." The doctor's wife and helpmeet is a good study of one who acted up to her convictions and faithfully supported her husband through the dark days when he became the victim of circumstances arising out of fidelity to what he, with those who "went out" with him, considered to be the plain leading of conscience.

"Mrs. Menzies was the daughter of a laird with a good pedigree and a fair estate, and the sister of a laird similarly situated. And, although she had thought fit to marry Dr. Archibald Menzies and to submit to his hiding his light under a bushel by not having sufficiently worldly ambition to take measures to be transferred from Rowanden to a larger parish with a stipend more in keeping with his wants and those of his family, and a congregation better qualified to value his learning and eloquence, that was not to say that she was to forget what was due to him and to herself, and to every child belonging to them, from Marjorie to the infant in the cradle."

Marjorie is the elder daughter of the Manse, who gives the title to the book. She is still at the village school, when she first appears on the scene.

"Marjorie of the Manse was dressed with great plainness, and in materials not one whit better than those worn by the small farmers'—even cotters' daughters. She was well grown, with a certain squareness and sturdiness of carriage and gait, which might become stateliness in later years. Her chestnut hair was in plaited loops tied up with brown ribbons so as to serve the double purpose of keeping it out of the way and preserving it in order. Her clear bright eyes were chestnut too, rather chestnut than hazel." She was growing up under the wing of her mother, who was acknowledged to be the high-minded queen of the parish and an example to all other wives and mothers within the bounds, to take the lead, lay down the law, and be as much of an authority in her own line as Dr. Menzies was in his. "Marjorie reflected such a nature. She was full of duties and obligations, not only to her father and mother and to the overflowing brood at the Manse, but to every solitary old man or woman, or waif of a child in Rowanden. Inevitably her cares lent her an amount of sedateness for her years, but it was a sedateness shot through with youthful buoyancy." With the family vicissitudes of the Menzies readers who follow them in "A Daughter of the Manse," especially if they have been born north of the Tweed, and know and are in sympathy with Scottish family life as related here, will find much that will interest them. There are difficulties besides religious ones to be met in the Menzies' family history, and the tracking of these to a final and not unhappy *dénouement* lends a different and rather exciting undertone to a story which might otherwise be monotonous. Commodore Menzies, the retired naval officer, brother of the minister, is a quaint character, and with his shy, reserved, yet withal on occasions brief and stern manner, gives a welcome relief to customs and characters parochial.

* "A Daughter of the Manse." By Sarah Tytler. John Long. 6s.

### Everybody's Opinion.

### THE UNTRAINED NURSE AS MIDWIFERY PUPIL.

"L.O.S." writes: Having read the letter written by "L.R.C.P." I can only arrive at one conclusion, and that is the untrained nurse referred to must be a most dishonourable, ungrateful, and dangerous woman, and one to be avoided by doctor and nurse. I know by experience that there are some women with a little knowledge and unbounded impudence, who think that they know everything, and the person in question is undoubtedly one of these. Unfortunately such people not only do themselves harm, but they spoil things for others who come after them.

"M. D." writes: The letter by "L.R.C.P." in your issue of July 29th, reveals a state of affairs which proves the difficulty that professional men have at the present day in dealing with nurses whose education and attributes are unsuited to the work they have associated themselves with. It will become a serious matter in the future if such women as those described by "L.R.C.P." are allowed to become qualified midwives, and be able to defy or act in opposition to the medical profession. The very principle of the Midwives Act—a well-meaning, but I fear impracticable idea—was to allow the benefits of skilled nursing under medical supervision to the poor lying-in woman! Here we have a woman—she hardly merits the noble name of "nurse"—turning against the doctor who helped her to gain her L.O.S., and doing him harm, and yet he has taught her, has helped her to an honourable means of livelihood, and enabled her to obtain a decent post at any time! It emphasises, indeed, the need of having, as "L.R.C.P." says, trained, educated, and disciplined women; but, above all, they must follow the golden rule, and possess the innate goodness of heart which should animate the "conversation" (in the old sense) and daily life of nurses.

### THE REPORT OF THE SELECT COMMITTEE ON NURSING.

"L. M. M." writes: In the report of the Select Committee appointed to consider the expediency of providing for the Registration of Nurses, the following recommendation is given under paragraph 19: "There should be an annual publication of the Register of Nurses. For this purpose the Central Body should make provision for striking off the Register the names of those nurses who have died or who have ceased nursing, and also of those nurses who, in the opinion of the Central Body, have been guilty of serious misconduct in the discharge of their duty or of moral delinquency." That the Register should be revised annually is obviously necessary, and it is quite just that those nurses who have shown themselves unfit to be in the profession should have their names removed, in the same way that a lawyer may get struck off the rolls. But I emphatically dissent from the view that, because a nurse, once fully trained and registered, has given up nursing, she should have her name removed from the Register. Take the case of the nurse who has finished her training and is then required at home for a year or two, perhaps owing to the death of a parent, and afterwards resumes her nursing. Or the case of the nurse who marries. If she should be left a widow in a few years' time and obliged to earn her own living, must she join the ranks of the unregistered, *i.e.* untrained? Or must she pay a fresh fee to be again placed on the Register? Either way, a very great injustice would be done to many fully trained and capable women. I think I am correct in saying that a doctor's name remains on the Medical Directory until his death, whether he carries on a practice or not, and it is only fair that a nurse of good character, once fully trained and registered, should be recognised as such to the end.

### NURSING ASSOCIATIONS IN NATAL.

"One Who Tried" writes from Durban under date of July 4: I see by the issue of June 10th which we received this week, that a nurses' association is about to be formed in Pietermaritzburg. I have neither seen nor heard anything about it from local papers or from friends. Five shillings per day is reasonable for this part of the world. I suppose that the association will have to rent a house and board the members. House rent is very high. We pay £7 per month for a five-roomed house. Then, there are working expenses and labour, all equally costly. There was a nurses' co-operation in Durban; it was not a success. I paid a year's subscription—10s. 6d.—but only got one very third-rate case. I objected to living in the co-operation home. One could get no rest as it was a bungalow of one floor and a general scramble seemed to pervade the place. I was glad to retire to my former lodgings where I had my meals with some regularity and quietness when disengaged. Three years ago I joined the Nurses' Co-operation in Johannesburg, paid a year's subscription—£1 1s.—and never got a case. A Pretoria doctor sent me to the Northern Transvaal to nurse a fever case. I was away about ten weeks. I naturally went to the Co-operation premises in Jeppe Street on my return, but there was no room for me. I had to go to one of the hotels which cost me £1 5s. per day. After a week of this, and there still being no room at the home, I thought I had better try Natal, as living in 1902 and 1903 was somewhat cheaper there than in Johannesburg. I remained here, but have given nursing homes and associations a wide berth ever since. I hope the new venture will be on different lines to those of which I have had experience.

### A BABY THREE WEEKS OLD AT A SCHOOL TREAT.

"Maternity Nurse" writes: Very recently I was spending a holiday at one of the well-known seaside resorts, and it became to me somewhat interesting to watch day-by-day for three weeks, all the excursions and school treats, and take note of how many mothers were present, also how many of them had taken more thought about joining the company and feeding on "ice creams," than about the discomfort of the long clothes babies they were carrying for the whole day in the heat. One mother particularly attracted my attention. She could not settle with her baby anywhere; at last she sat flat down on the sand to feed it; then the child was twisted and turned and shook—so much so that at last I went to her and said "give me your baby while you get up; how old is he?" She replied, "Three weeks, mum, I couldn't bring the gel, she's 13 months." I examined the baby as far as I could by putting its clothes straight, and I said, "Oh, my poor darling, what bad eyes," for the baby had ophthal[illegible]a badly, and the sun and the wind seemed too much for them. The mother said, "Yes, he's under the 'orspital for them." I begged her to take care of the eyes and told her the consequences of neglect. Placing the baby in her arms I felt that the body of the little thing was bound up so tightly that I feared before the end of the journey home to Onslow Square, London, he would be gasping for breath. Could not the clergy refuse to take children under a certain age to school treats?"

### AN INTERESTING CASE.

C. M. Heath, Boxmoor, Herts, writes: I am sending you a record of an interesting case I am attending, which I think may prove equally so to a section of the readers of your paper. On July 23rd last I was called to a woman at Boxmoor, and delivered her of twin sons, prematurely at seven months. The elder twin, William, weighed one and three-quarter pounds at birth—the second, Horace, two pounds and seven ounces. They are still alive and to all appearance doing very well—the smaller, William, having gained six ounces in weight during the week and Horace five ounces. There was no incubator available and I have fed the babies with their mother's milk in a spoon. In addition, I have given them three drops of brandy in a teaspoonful of boiled water with sugar twice during the 24 hours. I have also added sugar to every spoonful of milk, as I thought they required fattening. I have not bathed them but have thoroughly rubbed them all over twice daily with warmed olive oil, and kept a hot bottle at their feet. The greatest difficulty was, I found, to keep up the body warmth, and for several days I noticed, from the abdomen downwards, a great tendency to getting cold and quite stiff. I found the rubbing with warm oil of the most use for this. I called in a medical man to see the infants soon after birth, and he considered the case unique. The tenth day is now reached and mother and babies are all doing well, though I hope to continue the children under my care for some time to come. Perhaps my christening the babies had a beneficial effect on them, as we nurses always think a baby does better afterwards.

### Appointments.

No charge is made for announcements under this head, and we are always glad to receive and publish appointments. The information, to insure accuracy, should be sent from the nurses themselves, and we cannot undertake to correct official announcements which may happen to be inaccurate. It is essential that in all cases the school of training should be given.]

CHILDREN'S HOSPITAL, LOWER SYDENHAM.—Miss Eva Hill has been appointed charge nurse. She was trained at Leicester Infirmary, and has since been on the private nursing staff at the Hospital for Sick Children, Bloomsbury, sister at the Children's Hospital, Luton, and sister in charge of the Infirmary at the Princess Mary Home, Addlestone.

CREWKERNE COTTAGE HOSPITAL.—Miss Winifred Jones has been appointed matron. She was trained at the County Hospital, Taunton, and has since been staff nurse at the Accident Hospital, Newmarket, matron of Victoria Cottage Hospital, Abergavenny, and matron of Westminster Memorial Hospital, Shaftesbury; she has also done district work at Abergavenny. She holds the Apothecaries' Hall certificate for dispensing.

ISOLATION HOSPITAL, DAVENHAM, CHESHIRE.—Miss Cecil Bell has been appointed matron. She was trained at Sunderland Infirmary, where she has since been charge nurse. She has also been matron of Sunderland Borough Sanatorium and matron of the Memorial Hospital, Blythe.

MITFORD AND LAUNDITCH UNION WORKHOUSE INFIRMARY, GRESSENHALL.—Miss Millicent Plumb has been appointed superintendent nurse. She was trained at West Ham Union Infirmary, and has since been staff nurse at Derby Union Infirmary.

PRESTON, FULWOOD, AND LONGRIDGE HOSPITAL.—Miss M. E. Crosse has been appointed matron. She was trained at Norfolk and Norwich Hospital, and has since been matron of the Infants' Hospital, Hampstead, and lady superintendent of Poor Law Children's Homes at Birkenhead.

ROYAL NATIONAL HOSPITAL FOR CONSUMPTION, IRELAND.—Miss Margaret Brown has been appointed night superintendent. She was trained at Dundee Royal Infirmary, where she was afterwards sister, and she has also been sister at Ruchill Fever Hospital, Glasgow.

RURAL HOSPITAL, MALVERN.—Miss Eveline K. Hobday has been appointed charge nurse. She was trained at the District Infirmary, Ashton-under-Lyne, where she has since done sisters' holiday duty.

UNION INFIRMARY, FIR VALE, SHEFFIELD.—Miss Annie Henry and Miss Alice Radcliffe, have been appointed sisters. Miss Henry was trained at the Union Infirmary, Belfast, and has since been night superintendent at the Ulster Infirmary Hospital, Belfast, superintendent nurse at the Union Infirmary, Boilerborough, and charge night nurse at the Union Infirmary, Carrickfergus. Miss Radcliffe was trained at Chorlton Union Hospital, and has since been sister at Halifax Union Hospital, and night sister at Ashton-under-Lyne Union Infirmary.

### "The Hospital" Convalescent Fund.

THE Hon. Secretary begs to acknowledge with thanks the receipt of 7s. 6d. per the Travel Correspondent of THE HOSPITAL.

### Presentations.

CARSHALTON COTTAGE HOSPITAL.—A meeting of persons interested in the Carshalton Cottage Hospital was held on Friday evening at the West Street Schools to make a presentation to Nurse Unity Kinsman, who is resigning her post at the hospital. The testimonial consisted of a purse containing £40, with a list of the subscribers. Dr. Walter Gripper took the chair, and in making the presentation referred to the good work done by Nurse Kinsman in the hospital for the past five years, to the esteem in which she was held by all with whom she had come in contact, whether as patients or visitors, and the great regret felt at the enforced severance of a connection which had been so advantageous to the institution.

### Novelties for Nurses.

(BY OUR SHOPPING CORRESPONDENT.)

### CHOCOLATE AND COCOA FOR THE TRAVELLER.

THE wise traveller is he or she who provides against those unforeseen events which have a way of upsetting the most carefully thought out plans. The pedestrian and cyclist is especially prone to become a victim to the vagaries of fortune. A late arrival at some resting-place may be a small misfortune, but when the pangs of hunger are added to disappointment the matter is more serious. Therefore, those who are wise provide against such a mishap, and never venture afield without provisions. Messrs. Fry have reminded us that their excellent chocolate and cocoa specialities meet the requirements of every holiday-maker. Their milk chocolate is a form of concentrated food of the highest nutritive value, and excellent to allay hunger. It can be carried without adding a perceptible burden to the travelling kit. A tin of concentrated cocoa, or of the still more nourishing malted cocoa, renders the traveller independent of the unskilled and uncleanly cook. All that is needed to complete a repast is some bread and a little milk. These are nearly always obtainable when the larder is otherwise bare. Neither chocolate nor cocoa are spoilt by keeping, and no harm is done if the need for them does not arise on the journey. They can be stored for use on another occasion.

### TRAVEL NOTES AND QUERIES.

BY OUR TRAVEL CORRESPONDENT.

CONVENTS AT TROUVILLE (Nurse).—I know of no convents at Trouville. It is one of the most expensive places in France, entirely a *ville de luxe*, and unless money is of no consequence I should advise you to go almost anywhere else rather than to a very fashionable, but otherwise uninteresting, place, such as Trouville. I could tell you of convents in Normandy, Brittany, or Belgium. If you will write to me again at once, telling me what you can afford to spend and what your tastes are, I am sure I can tell you of some suitable place.

ACCOMMODATION IN PORTUGAL (Sister M.).—Many thanks for the address and kind information given. It is not often that my correspondents have money or time enough to go so far, but I have been asked several times for such a house as the one you speak of and I have gladly filed your letter.

ACCOMMODATION IN TURIN (Pseudonym forgotten).—The Travel Correspondent has lost the letter of the lady who has a boarding-house in Turin, and would be glad to have it again with particulars as to terms, etc.

A MONTH AT ST. MALO (A. M. F.).—I should discourage your living actually in the town of St. Malo; the town is squeezed within high ramparts which makes it airless; the smell from the dock is sometimes unpleasant, and the hotels are expensive and far beyond your means. Mrs. Dyott, Villa Olga, Paramé, and Mrs. Wise, 19 Rue Darnyean, St. Servan, have houses that would suit you; both places are close to the old town. The excursion tickets to St. Malo from Southampton are only for a fortnight's stay.

### Notes and Queries.

### REGULATIONS.

**The Editor is always willing to answer in this column, without any fee, all reasonable questions, as soon as possible.**

**But the following rules must be carefully observed.**

**1. Every communication must be accompanied by the name and address of the writer.**

**2. The question must always bear upon nursing, directly or indirectly.**

**If an answer is required by letter a fee of half-a-crown must be enclosed with the note containing the inquiry.**

*Nursing in Hop Gardens.*

(152) Will you kindly tell me where to apply, and when, for work amongst the hop-pickers this summer? Also any information, if possible, about salary usually given.—*Nurse L. L.*

Write to Miss Harvey, 265 Vauxhall Bridge Road, London, S.W. You should apply at once, as hop-picking will probably commence the last week in August. No remuneration is given, but board, lodging, and travelling expenses are provided.

*Llandudno.*

(153) Can you tell me the address of any holiday home for nurses in Llandudno?—*M. B.*

You might write to the Secretary of the Sanatorium, 5 Clonmel Street, Llandudno, N. Wales.

*Massage.*

(154) Will you kindly tell me if there is any place in Manchester where I could receive a course of instruction in massage?—*M. E. T.*

Write to the Secretary, The Manchester School of Massage, 24 Lime Grove, Longsight, Manchester.

*Poor-law Certificate.*

(155) Will you kindly let me know if a Poor-law certificate prevents me from entering Queen Alexandra's Imperial Military Nursing Service or the Indian Nursing Service, also where to get particulars of the latter?—*Inquirer.*

For full particulars of the conditions of entering each Service, write to the Secretary, the War Office, 68 Victoria Street, London, S.W.

*Training.*

(156) Will you kindly inform me which are considered good training schools for nurses, and also what premiums are required, or could you advise me how to obtain that information? Are any of the ——— hospitals good schools?—*E. M.*

See "The Nursing Profession: How and Where to Train." The schools you mention are good.

(157) Do you think that 33 is too old to enter as probationer nurse? I have had two years' mental training, but would like to go to a hospital.—*N. S.*

At your age you would find it difficult to train unless you went as a paying probationer.

*Male Nurse.*

(158) I am rather dubious as to which course to follow for the best. My age is 23 and I have had two years' asylum nursing experience, and as I wish to go in for private nursing, do you advise me to stay another 12 months and get the Medico-Psychological certificate and keep to mental nursing? Or do you think that it would pay me better to have, say, two or three years' training at a hospital where they train male nurses? If so, at what hospital should I apply?—*W. L.*

We should advise you to go in for the Medico-Psychological certificate and keep to mental nursing if you like the work, as it is remunerative, and the demand in England for male nurses for general cases is not great. But if you decide not to go on with your mental work and wish for other training, write to the matron of the National Hospital for the Paralysed and Epileptic, Queen Square, London, W.C., or try the Army Medical Corps. The address of the latter is 68 Victoria Street, S.W.

*Hysteria.*

(159) Will you kindly inform me if there is any home for hysteria subjects on payment of small sum monthly?—*E. B.*

Hysteria is a disease and must be prescribed for and treated by a medical man who will probably be able to recommend a home.

**Handbooks for Nurses.**

| | Post Free. |
|---|---|
| "How to Become a Nurse: How and Where to Train." | 2s. 4d. |
| "Nursing: its Theory and Practice." (Lewis.) ... | 3s. 6d. |
| "Nurses' Pronouncing Dictionary of Medical Terms." ... | 2s. 0d. |
| "Complete Handbook of Midwifery." (Watson.) ... | 6s. 4d. |
| "Preparation for Operation in Private Houses." ... | 0s. 6d. |

Of all booksellers or of the Scientific Press, Limited, 28 & 29 Southampton Street, Strand, London, W.C.

### For Reading to the Sick.

### THE SUN BEHIND THE CLOUD.

It is easy enough to be pleasant
When life flows by like a song;
But the man worth while is the one who will smile
When everything goes wrong.
For the test of the heart is trouble,
And always comes with years;
And the smile that is worth the praises of earth
Is the smile that shines through tears.

*Anon.*

The secret of Christian joy is the peace of Christ in the heart. Then one is not dependent on circumstances or conditions. St. Paul said he had learned in whatsoever state he was, therein to be content. That is, he had formed a habit of happiness and had mastered the lesson so well, that in no state or condition, whatever its discomforts were, was he discontented. We know well that his circumstances were not always congenial or easy. But he sang songs in his prison with just as cheerful a heart and voice as when he was enjoying the hospitality of some loving friend. His word was always one of cheer, not only when things went well, but when things went adversely. He was just as songful on his hard days as on comfortable days.

The power to hear what Nature's voices have to say is in the heart, not merely in the ear. We must have the beauty in our soul before we can see beauty anywhere. Hence there are many who are really blind to the loveliness which God has shown everywhere, with most lavish hand in his works. So we must have the music in our heart before we can hear the music which sings everywhere for him who has ears to hear. If we have thanksgiving within us we will have no trouble in finding gladness wherever we go. It is a sad cheerless heart that makes the world dreary to certain people; if only they would let joy enter to dwell within, a new world would be created for them.

There is a legend of a wonderful bell that rings in Heaven, whose sweet tones only those can hear whose hearts are pure and gentle. If we allow our hearts to cherish unlovingness, bitterness, evil thoughts and feelings, we cannot hear the music of love which breathes everywhere pouring out from the heart of God. But if we keep our heart gentle, patient, lowly, and kind, on our ears will fall wherever we go, sweet strains of divine music out of heaven.—*J. R. Miller.*

It is said somewhere, at twilight
A great bell softly swings,
And man may listen and hearken
To the wondrous music that rings.

If he puts from his heart's inner chamber
All the passion, pain and strife,
Heartache, and weary longing,
That throb in the pulses of life;

If he thrusts from his soul all hatred,
All thoughts of wicked things,
He can hear in the holy twilight
How the bell of the angels rings.

*Anon.*

## Figures and Tables

**Fig. 5. f1:**
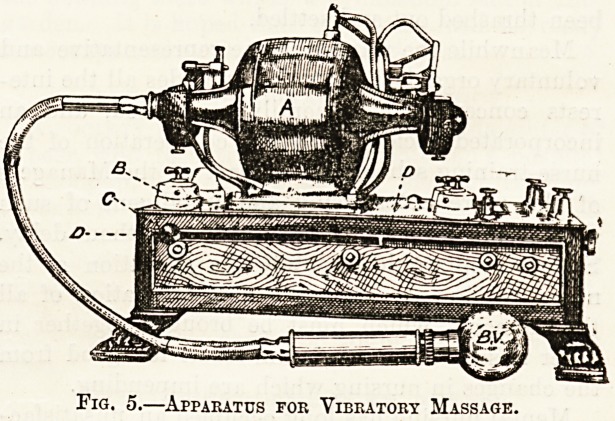


**Fig. 6. f2:**
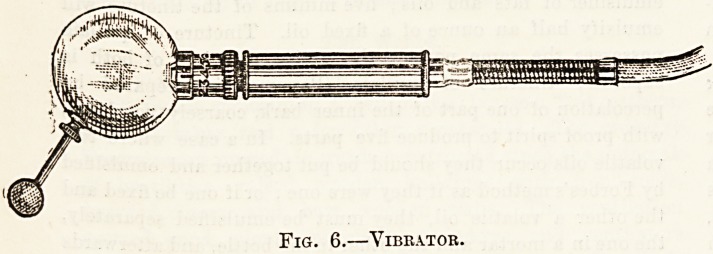


**Figure f3:**